# Impact of Spinal Cord Injury on Mental Health: A Narrative Review

**DOI:** 10.7759/cureus.100422

**Published:** 2025-12-30

**Authors:** Katerina Gklantzouni, Dimitrios-Stergios Evangelopoulos, Maria-Eleftheria Evangelopoulos, Spiridon Pnevmaticos

**Affiliations:** 1 Postgraduate Training Program, KAT Hospital, National and Kapodistrian University of Athens School of Medicine, Athens, GRC; 2 3rd Department of Orthopedic Surgery, KAT Hospital, National and Kapodistrian University of Athens School of Medicine, Athens, GRC; 3 Laboratory for Research of the Musculoskeletal System “The Garofalidis”, KAT Hospital, National and Kapodistrian University of Athens School of Medicine, Athens, GRC; 4 1st Department of Neurology, Eginition Hospital, National and Kapodistrian University of Athens School of Medicine, Athens, GRC

**Keywords:** anxiety, depression, mental health, psychosocial factors, quality of life, spinal cord injury

## Abstract

Spinal cord injury is a condition that radically alters an individual’s life, significantly affecting their mental health. This narrative review synthesizes evidence on depression, anxiety, post-traumatic stress disorder (PTSD), and suicidal ideation after spinal cord injury and reports key factors associated with poorer or more favorable mental health outcomes, to inform rehabilitation strategies and improve quality of life.

A literature search was conducted in PubMed and Scopus, covering the last decade (2015-2025). Thirty studies met the predefined eligibility criteria. The main outcomes examined were depression, anxiety, PTSD, suicidality, and overall quality of life linked to mental health. Outcomes were assessed using validated instruments reported in the included studies (e.g., Patient Health Questionnaire-9 (PHQ-9), Generalized Anxiety Disorder-7 (GAD-7), and Hospital Anxiety and Depression Scale (HADS)).

Across the included studies, individuals with spinal cord injury generally reported higher prevalence rates compared with general population estimates for depression (26%-35%), anxiety (10%-26%), PTSD (12%-36%), and suicidal ideation (11%-33%). Factors correlated with poorer mental health outcomes included socioeconomic disadvantages, pain interference, and secondary health conditions. In contrast, psychosocial factors such as resilience, self-efficacy, and social support, as well as physical activity, were associated with better psychological outcomes.

This narrative review highlights the substantial impact on mental health that has been reported after spinal cord injury, with higher rates of depression and other psychiatric disorders compared to the general population. By summarizing factors associated with poorer and more favorable mental health outcomes, this review aims to inform rehabilitation programs with a holistic approach, support timely identification of mental health issues, and guide management approaches to promote quality of life among individuals with spinal cord injury.

## Introduction and background

Spinal cord injury (SCI) is a life-altering condition that often leads to permanent impairments in motor, sensory, and autonomic functions. Most cases involve traumatic SCI, typically resulting from external causes such as road traffic accidents, falls, or acts of violence, with iatrogenic causes being rare. In contrast, non-traumatic SCI is less common and may arise from vascular disorders, neoplasms, congenital anomalies, exposure to toxins, etc. According to the World Health Organization (WHO) [1], more than 15 million individuals globally are living with SCI, with an estimated annual incidence of 23.77 cases per million population [2].

This debilitating condition is associated with severe physical complications, such as respiratory, cardiovascular, urinary, and bowel complications, musculoskeletal disorders, spasticity, pain syndromes, pressure ulcers, autonomic dysreflexia, and osteoporosis [3,4], that significantly increase mortality rates. In addition to the somatic burden, SCI also imposes substantial psychological and emotional challenges. According to recent studies, the prevalence of mental health disorders among individuals with SCI is substantially higher than that in the general population. Adults with SCI have been found to exhibit a markedly greater incidence of any psychological morbidity (59.1% vs. 30.9%) compared with adults without SCI [5]. Specifically, the prevalence of depression in this population is estimated at 22.2% with a wide range of 9.8%-37.5% based on a recent meta-analysis [6,7], whereas the corresponding prevalence in the general population is approximately 5.7%, as reported by the WHO [8]. In addition to depression, individuals with SCI frequently experience other mental health conditions, such as anxiety, post-traumatic stress disorder (PTSD), substance use disorder, suicidal ideation, and reduced life satisfaction. These psychological sequelae often persist well beyond the acute phase of injury, potentially hindering rehabilitation, diminishing quality of life (QoL), and adversely affecting long-term health outcomes.

Several factors may contribute to the elevated prevalence of mental health disorders following SCI. The abrupt loss of mobility and independence, coupled with chronic pain and the necessity to adapt to significant physical limitations, can profoundly disrupt an individual’s sense of identity and autonomy. Changes in social and occupational roles often compound the psychological impact, as individuals may face reduced participation in community life and limited employment opportunities. Furthermore, environmental, socioeconomic, and personal factors-including access to appropriate healthcare services, availability of social support, pre-existing mental health status, and resilience-play a critical role in shaping psychological adjustment after SCI. These interrelated factors not only influence the onset of mental health disorders but may also determine the severity and persistence of such conditions over time.

Mental health after SCI has received increasing research attention in recent years; however, it has not yet been studied comprehensively or with sufficient consistency, particularly when compared with the physical aspects of rehabilitation. Understanding the mental health burden in individuals with SCI is essential for improving current clinical management and for developing a rehabilitation model with a holistic approach, addressing both the physical and psychological changes that occur following the injury. Although numerous studies have examined mental health outcomes in this population, the evidence is often fragmented across different disciplines and methodological approaches.

Therefore, this narrative review aims to investigate the existing literature on the impact of SCI on mental health, with a focus on identifying common psychological morbidities and factors associated with poorer and more favorable mental health outcomes. It is anticipated that our findings will contribute to the early identification and targeted management of psychological difficulties. Such efforts may support the optimization of healthcare delivery and, ultimately, improve the QoL for individuals living with SCI.

## Review

Study design

This study was conducted as a narrative review aiming to synthesize current evidence on the mental health consequences of SCI, with particular attention to depression, anxiety, PTSD, suicide risk, substance use disorders, psychological well-being, and overall QoL in affected individuals. A narrative review approach was chosen because the existing literature on mental health outcomes after SCI is highly heterogeneous in terms of study design, populations, and outcome measures, making quantitative meta-analysis inappropriate. Although several systematic elements were incorporated (structured electronic search, predefined eligibility criteria, explicit screening procedures, standardized data extraction, and the use of a flow diagram to summarize study selection), PRISMA (Preferred Reporting Items for Systematic reviews and Meta-Analyses) 2020 reporting guidelines were not formally applied given the narrative design [9]. This work is best classified as a narrative review due to the absence of protocol registration, the relatively limited search strategy, and the primarily descriptive approach to synthesis.

Literature search strategy

A literature search was performed in two electronic databases, PubMed and Scopus. The keywords “spinal cord injury” AND “mental health” were used as search terms. The search was limited to articles published within the last 10 years (2015-2025). Additional filters were applied to restrict the results to studies published in English and available as open access. For the Scopus database, an additional filter was set to include only studies categorized under Medicine. For transparency, the database-specific search strategy (terms and filters) is provided in Appendix A. In addition, the reference lists of several key articles identified through the electronic database searches were screened to identify further relevant studies that could inform the background and interpretation of this review, although this citation tracking was not conducted systematically for all included papers.

Eligibility criteria

Inclusion and exclusion criteria specified the population, intervention/exposure, comparator, outcomes, and study designs considered. Studies were eligible for inclusion if they investigated adults (≥18 years) with traumatic or non-traumatic SCI, including individuals with paraplegia or tetraplegia, in community or rehabilitation settings. Eligible studies were required to report at least one quantitatively assessed mental health outcome after SCI, such as depression, anxiety, PTSD, suicidal ideation, or substance use disorder, using a validated instrument. Measures of resilience or life satisfaction were also considered when explicitly analyzed in relation to mental health. We included primary quantitative research studies, such as randomized controlled trials (RCTs), cohort studies, cross-sectional investigations, and large registry-based analyses. No restrictions were applied regarding the intervention or exposure context, provided that mental health outcomes were assessed.

Exclusion criteria were applied to studies focusing on children or adolescents, caregivers, or healthcare professionals, as well as those addressing neurological conditions without SCI-specific analysis. We excluded studies examining only pain, fatigue, sleep, physical rehabilitation, or general QoL in the absence of specific mental health outcomes. Purely qualitative research, validation studies of psychometric tools, pilot studies, protocols, case reports, systematic reviews, meta-analyses, guidelines, and commentary papers were also excluded. Only articles published in English with full-text availability were considered.

Study selection and data extraction

All titles and abstracts identified through the database searches were initially screened by the first author (K.G.) against the predefined eligibility criteria. A second, senior reviewer (D.E.) independently verified all screening decisions. The full texts of potentially eligible articles were then assessed in the same way, with one reviewer performing the initial assessment and a second reviewer verifying inclusion and exclusion decisions. Disagreements were resolved through discussion and consensus between the two reviewers and, when needed, in consultation with a third team member (M.E.). The same process was followed with data extraction, performed by a single reviewer (K.G.) and independently verified by a second reviewer (D.E.), using a structured data extraction form (Appendix B).

The following variables were collected from the included studies: first author, year of publication, journal, country, study design, study objective, title, and participant characteristics (e.g., sample size, age, sex, injury level and severity, and time since injury). Information on mental health outcomes assessed (depression, anxiety, PTSD, suicidal ideation, substance use, resilience, and life satisfaction) and the methods or instruments used to measure these outcomes were also extracted. Representative instruments included the Patient Health Questionnaire-9 (PHQ-9) [10], Generalized Anxiety Disorder-7 (GAD-7) [11], Hospital Anxiety and Depression Scale (HADS) [12], Beck Depression Inventory (BDI) [13], Center for Epidemiologic Studies Depression Scale (CES-D) [14], State-Trait Anxiety Inventory (STAI) [15], and the Posttraumatic Stress Disorder Checklist for DSM-5 (PCL-5) [16]. A complete list of instruments, together with the assessment time frame and cut-off/case definition (when reported), is provided in Appendix C. We also extracted information on whether the instruments used had been validated in individuals with SCI and whether validated translations in the local language were applied.

Where applicable, data on subgroup comparisons (e.g., public vs. private healthcare, socioeconomic differences, or intervention vs. control groups) were recorded. Extracted data were compiled into evidence tables and organized thematically according to the mental health outcomes of interest. Special cases, such as the impact of the COVID-19 pandemic and differences between veterans and the general SCI population, were also noted.

Data synthesis

Due to heterogeneity of the included studies in study design, population, and outcome measures, a meta-analysis was not feasible. Instead, a narrative synthesis was undertaken. Findings were organized thematically into three domains: (1) prevalence of mental health disorders (depression, anxiety, PTSD, suicidality, and QoL), (2) factors associated with poorer and more favorable mental health outcomes (including socioeconomic status, pain, resilience, and coping strategies), and (3) intervention findings assessing the impact of rehabilitation or targeted therapies on psychological outcomes. Extracted data were summarized in evidence tables and synthesized narratively, highlighting both consistencies and discrepancies across studies.

Quality assessment

The methodological quality of this narrative review was assessed using the SANRA (Scale for the Assessment of Narrative Review Articles) [17], which includes six items rated from 0 to 2 (0 = not fulfilled, 1 = partially fulfilled, and 2 = fully fulfilled; maximum total score 12). Two authors (K.G. and S.P.) independently rated all items and resolved any disagreements by discussion. The completed SANRA checklist is provided in Appendix D.

Quality assessment of the included studies

The methodological quality of the included studies was assessed using a predefined light-touch seven-domain checklist developed by the author team for this narrative review. For each study, we evaluated (1) clarity of the research objective; (2) appropriateness of the study design for the stated research question; (3) description of the study population, including eligibility criteria and key participant characteristics; (4) measurement quality, including the use of validated mental health instruments and clear definition of SCI characteristics (e.g., injury level/severity); (5) clarity and appropriateness of the data analysis, including reporting of measures of uncertainty (e.g., confidence intervals, p-values); (6) consideration of potential sources of bias and confounding (e.g., age, sex, comorbidities, and missing data); and (7) transparency regarding funding sources and conflicts of interest.

Each domain was rated as Yes, No, Partial, or Unclear. As an overall summary, studies with Yes ratings in ≥6 domains were considered high quality, those with Yes in 4-5 domains were considered moderate quality, and those with Yes in ≤3 domains were considered low quality. Quality assessment was initially performed by one reviewer (K.G.) and independently checked by a second, senior reviewer (D.E.); any disagreements were resolved through discussion, with arbitration by a third senior author (S.P.) when necessary. The study-level quality assessment of the included studies using the seven-domain checklist is provided in Appendix E.

Ethical considerations

This study is a narrative review of published research on mental health outcomes after SCI. No administration, reproduction, or redistribution of instrument items or scoring materials was performed; instruments are mentioned by name and cited to their sources. Free-to-use instruments (e.g., PHQ-9 [10], GAD-7 [11], CES-D [14], and PCL-5 [16]) require no license, whereas copyrighted instruments (e.g., HADS [12], BDI [13], and STAI [15]) are cited without reproducing content. No human participants or animals were involved; therefore, ethical approval and informed consent were not required. The review adhered to accepted standards of research integrity and reporting.

Results

The initial search retrieved a total of 2,674 records (PubMed = 1,241; Scopus = 1,433). After applying predefined filters (publication years 2015-2025, English language, open access, and “Medicine” subject area in Scopus), 980 articles were retained (PubMed = 530; Scopus = 450). Following the removal of 180 duplicates, 800 records remained for screening. Titles and abstracts were screened, leading to the exclusion of 652 articles that did not meet the eligibility criteria.

**Figure 1:**
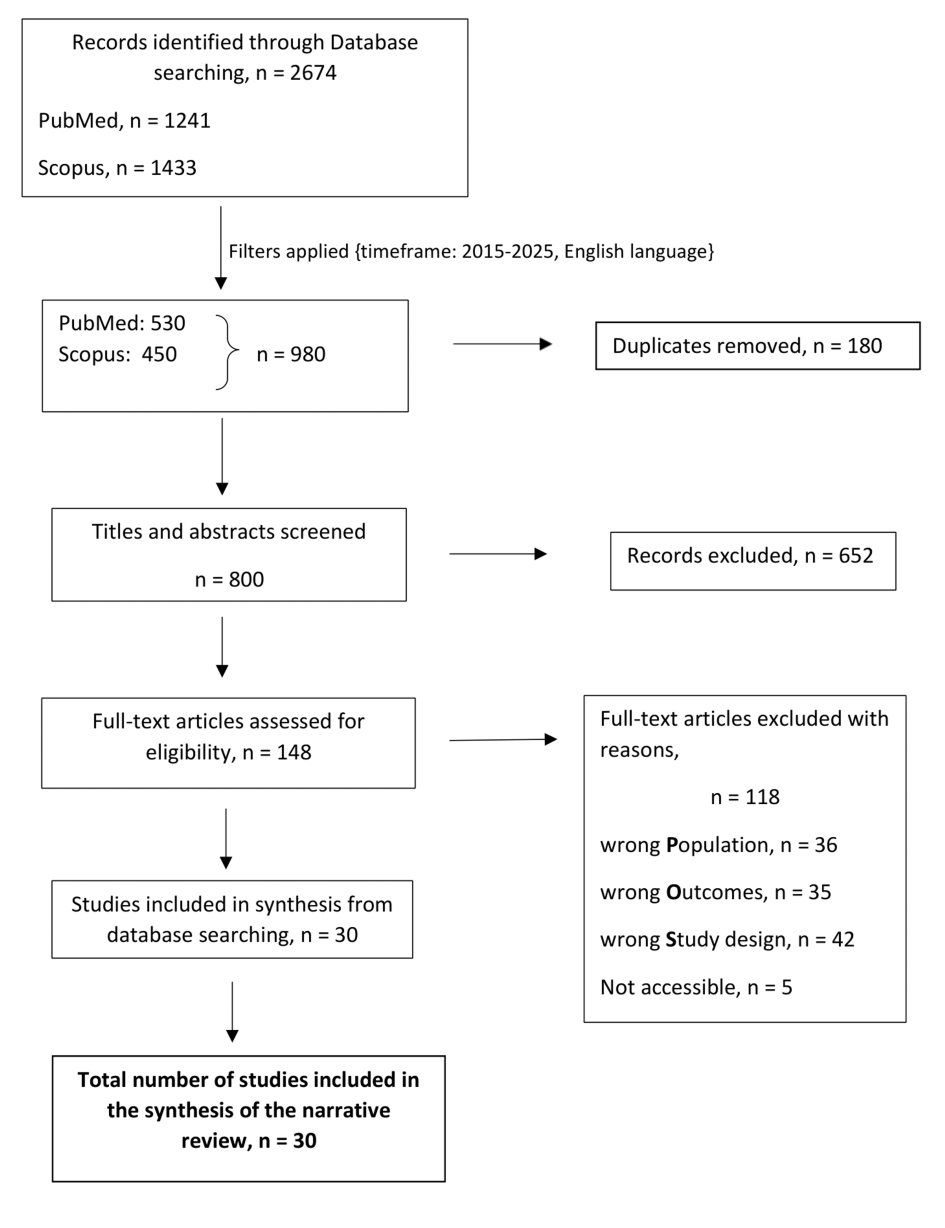
Flow diagram of the study selection process for this narrative review This diagram summarizes the study selection process; PRISMA guidelines were not formally applied due to the narrative review design [9]. PRISMA: Preferred Reporting Items for Systematic reviews and Meta-Analyses

A total of 148 full-text articles were assessed in detail, of which 118 were excluded. Reasons for exclusion included studies focusing on caregivers, other neurological conditions, purely qualitative designs, psychometric tool development, protocols, case reports, reviews, and studies addressing only pain, functioning, or general QoL without direct assessment of mental health outcomes. Ultimately, 30 studies were included in the final synthesis. The study selection process is illustrated in Figure 1 (flow diagram).

Thirty studies met the inclusion criteria. Sample sizes ranged from 51 participants to very large population datasets (>40,000 and ~12,500 participants) [18,19], with most studies enrolling between 51 and 504 individuals and three mid-sized cohorts at n = 940, n = 1,549, and n = 3,136 [20-22]. Most samples were male-predominant (55%-98.6% male), although one national survey reported a more balanced sex distribution (47% male) [18]. Mean ages typically fell between 32 and 59 years, with one study spanning 16-75 years [22]. Both paraplegia and tetraplegia were represented. Time since injury varied from acute rehabilitation through long-term community cohorts (up to 32 years post-injury). The studies were conducted across diverse regions, including the United States (n = 14) [20,22-34], Canada [18,22], Spain [35], Sweden [21,36,37], Switzerland [38], the Netherlands [39], Poland [40], Turkey [41], South Korea [42], Nepal [43], India [44], South Africa [37,45], and Australia [46,47]. In addition, one large multi-country survey contributed data from 22 countries [19]. Study designs were primarily cross-sectional (n = 17), alongside longitudinal cohorts (including two inception cohorts) [20,27-29,33,38,39,46], retrospective/registry analyses [18,22], two prospective intervention studies [40,47], and one RCT [26].

Regarding the results of the quality assessment, the SANRA [17] rated the overall methodological quality of this review as 10/12, indicating high methodological quality for a narrative review. Using the light-touch seven-domain quality appraisal of the included studies, 23 studies were rated as high quality [18-21,23,25-28,30-34,36-39,41,42,44,46,47] and seven as moderate quality [22,24,29,35,40,43,45]. Among the high-quality studies, 16 achieved a score of 6/7, and seven achieved a full score of 7/7. No studies were rated as low quality. The main limitations observed were related to cross-sectional design and incomplete reporting of confounder control.

Depression

Depression was the most frequently reported outcome, assessed in 27 studies, and was commonly measured using validated tools (e.g., PHQ-9, HADS-D, and BDI) [10,12,13]. Across community and rehabilitation settings, most studies reported that 16%-35% of participants screened positive for clinically important depressive symptoms, reflecting period prevalence (most often the past two weeks or past month, where reported) [18,20,21,24,25,27,32,35-37,39,42]. Higher rates were associated with chronic injuries [20,27,28,35,36], high pain interference [21,27,28,31,33,45], incomplete injuries [23,24,36], secondary health conditions (SHCs) [21,22,25,37,38,46], a history of preinjury mental health problems [27], and socioeconomic disadvantage [18,19,23,37,38,44]. A notable outlier was a community study from Nepal, in which approximately 60.8%-84.3% screened positive for depressive mood; this pattern may reflect local conditions, including limited access to formal mental health services and socioeconomic hardship in low-income settings [43]. Longitudinal evidence suggested that depressive symptoms may persist beyond discharge, with distinct trajectory subgroups (e.g., consistently low vs. persistently elevated), and greater depressive symptom severity over time was correlated with higher pain interference [18,27,28,33]. Factors associated with more favorable depressive symptom outcomes included higher resilience [23,29], self-efficacy [39,46], social support [29], and engagement in physical activity [21,22,30,36,42].

Anxiety

Anxiety, typically assessed using instruments such as the HADS [12], GAD-7 [11], or STAI [15], was measured less frequently than depression but remained a common outcome across 15 studies [18,20,22,23,25,29,31,32,35,37-39,40,42,45]. Reported anxiety prevalence estimates, reflecting point or period prevalence when specified, ranged from 10% to 26% [18,25,29,35] and frequently co-occurred with depressive symptoms [18,20,35]. Factors associated with higher anxiety included insomnia severity [25], low resilience [23], financial strain [18], high pain interference [31,45], and obesity [22]. In contrast, lower anxiety was associated with physical activity [22,36,42] and higher self-efficacy; the latter was associated with membership in lower-anxiety trajectory groups during the first year post-injury [39,47].

PTSD and Suicidal Ideation

PTSD [24,25,34,41] and suicidal ideation [18,32,41] were examined in fewer studies but remained clinically important mental health concerns following SCI. For PTSD, reported prevalence estimates ranged from 12% to 36% [24,25] and primarily reflected screen-positive PTSD symptoms over a defined period (most commonly the past month, when reported). Several factors were associated with higher PTSD symptom burden and poorer psychological adjustment, including event centrality, a cognitive appraisal variable, describing the extent to which SCI becomes a central life event that disrupts identity [34]. In addition, studies conducted in veteran cohorts reported an association between veteran status and PTSD symptoms. These findings reflect evidence from a distinct veteran population and may not generalize to non-veteran SCI samples; some authors suggested this could relate to higher preinjury or service-related traumatic exposure [24,25].

Suicidal ideation was reported in a meaningful minority of participants and was often observed alongside depression and PTSD [18,41]. Prevalence estimates ranged from 11% to 33%, most frequently assessed using PHQ-9 item 9 [10], which captures suicidal thoughts over the prior two weeks and therefore reflects short-period prevalence [32,41]. Suicidal ideation was consistently associated with depression [32,41] and was also associated with socioeconomic hardship [18] and reduced life satisfaction [24,32]. In contrast, greater resilience and physical independence were correlated with more favorable outcomes [32,41].

Other Mental Health Outcomes

Evidence on alcohol and substance use was limited. In one study of U.S. veterans with SCI, alcohol use disorder was reported in 8% of participants and substance use disorder in 5.3% [24].

Psychological and Socioeconomic Correlations

Among the most consistent findings across outcomes were modifiable psychosocial resources, such as resilience [23,29], self-efficacy [39,46], adaptive coping strategies [36], and social support [31,38,43], which were associated with fewer depressive and anxiety symptoms and greater life satisfaction. Socioeconomic gradients were also apparent: financial strain was correlated with poorer mental health outcomes, including higher depressive symptom burden [16,17,21,36]. Education and income showed weaker associations [19,23], whereas higher socioeconomic status and employment were associated with better psychological QoL [44]. A study by Joseph et al. in South Africa, comparing individuals treated in the public versus private healthcare sectors, found that psychological distress remained high in both groups, highlighting that mental health burden may persist even across different levels of healthcare access and resources [37]. At the macro level, country-level socioeconomic development did not fully account for differences in mental health outcomes, underscoring the potentially important role of social and cultural factors [19].

Pain, Participation, and SHCs

Higher pain interference [21,28,31,33,36,45], obesity [22], insomnia severity [25], and other SHCs [21,36,37,47] were associated with poorer mental health outcomes. Participation and community engagement were associated with higher life satisfaction and lower depressive symptom burden [21,31,38,43], and in several studies, these factors were examined as potential moderators of the association between pain interference and depression [21,31,38,43]. In studies including veterans and in long-term cohorts, perceived understanding from others and social support were correlated with more favorable mental health profiles [24,25,31].

Impact of the COVID-19 Pandemic on Mental Health

Two U.S. studies conducted during the COVID-19 pandemic in 2020 reported elevated depressive and anxiety symptoms among individuals with SCI, in the context of reduced access to services and limited community participation. Higher resilience was associated with lower psychological distress [23,29].

Intervention Findings

Evidence from intervention studies was limited but generally encouraging. One RCT found that, compared with usual care, collaborative care was associated with reduced depressive symptom severity, higher life satisfaction, and greater adherence [26]. In addition, a prospective clinical study of robotic-assisted gait therapy reported pre-post improvements in depression and anxiety; however, these changes were comparable to those observed with conventional therapy, and potential confounding could not be excluded [40].

Summary

Despite methodological heterogeneity, several coherent patterns emerged. Depression and, to a lesser extent, anxiety, PTSD, and suicidal ideation were consistently reported as common outcomes following SCI. Poorer mental health outcomes were associated with pain interference, SHCs, and socioeconomic disadvantage. Conversely, resilience, self-efficacy, participation, and social support were correlated with lower psychological distress and more favorable well-being indicators. Outcome-level summaries and key associated factors are presented in Table 1.

**Table 1:** Mental health outcomes after SCI: prevalence/severity and factors associated with poorer and more favorable outcomes Depression was assessed in studies [18,20-27,29-34,35-39,40-43,45-47]. Anxiety was assessed in studies [18,20,22,23,25,29,31,32,35,37-40,42,45]. PTSD was assessed in studies [24,25,34,41]. Suicidal ideation was assessed in studies [18,32,41]. Resilience and QoL were assessed in studies [18,20,21,23,24,26,28-32,38,40,44]. AIS-D: American Spinal Injury Association (ASIA) Impairment Scale grade D: motor incomplete [48]; PTSD: post-traumatic stress disorder; QoL: quality of life; SHCs: secondary health conditions; SCI: spinal cord injury

Outcome	No. of studies	Prevalence/severity	Factors associated with poorer mental health outcomes	Factors associated with more favorable mental health outcomes
Depression	27	16%-35% (period prevalence); moderate to severe depression	Pain, secondary health conditions, low socioeconomic status, unemployment, social isolation, COVID-19 pandemic, obesity, veterans, less severe injury (AIS-D)	Resilience, self-efficacy, social support, physical activity, collaborative care, acceptance as a coping strategy, higher sense of coherence, employment
Anxiety	15	10%-26% (point or period prevalence), mild to very high severity	Low resilience, insomnia severity, secondary health conditions, pain interference, socioeconomic stressors, COVID-19 pandemic	Physical activity, higher self-efficacy
PTSD	4	12%-36% (period prevalence)	Event centrality, insomnia, comorbid depression	Resilience, social support
Suicidal ideation	3	11%-33% (period prevalence)	Depression, socioeconomic hardship, lower life satisfaction, reduced physical independence	Resilience, physical independence
Resilience/QoL	14	Higher resilience/better mental health and QoL	Pain, low participation, social isolation, multiple SHCs	Strong coping strategies, community integration, physical activity-leisure time

Discussion

How well do we truly understand the psychological burden of SCI beyond its physical consequences? The evidence synthesized in this review underscores the substantial mental health burden reported among individuals with SCI. Compared with general population estimates, the reported prevalence of depression, anxiety, and suicidal ideation appears higher. At the same time, consistent associations of resilience, adaptive coping strategies, and social participation with more favorable mental health outcomes point to potentially promising avenues for early identification and rehabilitation planning. The following section discusses the key mental health outcomes in detail, beginning with depression, which was by far the most consistently examined condition.

Depression

Depression was the most consistently reported mental health outcome following SCI, assessed in 27 included studies. Across these studies, prevalence estimates predominantly reflected period prevalence (most commonly the past two weeks or the past month, when reported) and ranged from 16% to 35% [18,20,21,24,25,27,32,35-37,39,42]. Although these estimates are not directly comparable with general population estimates due to heterogeneity in assessment methods and time frames, they appear higher than the WHO estimate of 5.7% depression prevalence in the general adult population (point/current estimate) [8]. These findings align with earlier work by Williams and Murray showing depression as the most common psychiatric complication after SCI [7]. Notably, one study from Nepal by Baniya et al. reported rates exceeding 60% and up to almost 85%, which may reflect the association of depressive symptoms with socioeconomic disadvantage and limited access to rehabilitation services in low- and middle-income settings [43]. The main factors that were repeatedly associated with depression across the included studies were pain interference [21,28,31,45], SHCs [21,36,37,46], obesity [22], less severe injuries (American Spinal Injury Association (ASIA) Impairment Scale grade D (AIS-D)) [23,24,36,48], social isolation [23,38], and low socioeconomic status [19,45]. Financial strain was independently associated with both general mental health problems and depressive symptoms [38]. Higher Insomnia Severity Index scores were related to greater depressive symptom severity, explaining an additional 19% of the variance in depressive symptoms in that study’s model [25]. Moreover, Molina-Gallego et al. reported a higher prevalence of depressive symptoms among individuals in the chronic stage of injury and among those with traumatic injuries [35].

A factor linked to more favorable mental health outcomes was resilience [23,29]; higher resilience was consistently associated with lower depressive symptom burden. This aligns with the findings of Min et al. [49], who reported that resilience independently contributed to reduced depressive symptoms and greater post-traumatic growth. Other protective factors included higher self-efficacy [39,46], social support and participation [31,38,44], sense of coherence [36], and employment [36,44]. Physical activity was also correlated with higher life satisfaction and may be associated with fewer depressive symptoms [21,30,36,42].

Anxiety

Anxiety was another frequently reported mental health outcome following SCI, although it received less consistent attention than depression, being examined in 15 studies. Across these studies, prevalence estimates reflected point or period prevalence (when reported) and ranged from 10% to 26% [18,25,29,35]. These estimates were generally lower than those for depression, and anxiety frequently co-occurred with depressive symptoms. Anxiety prevalence in SCI appears higher than the WHO estimate of 4.4% global current/point prevalence in the general population [50]. We note that lifetime prevalence estimates (reported as up to 28.8% in the general population) [51] are not directly comparable with the predominantly point/period estimates observed in the SCI literature. Higher rates were observed among veterans [25,31], individuals with comorbid insomnia [25], and those reporting persistent pain interference [31,45]. These figures correspond with prior systematic reviews indicating that elevated anxiety levels are common in SCI populations and often co-occur with depressive symptoms [7,52].

Several studies in this review also reported correlates of anxiety, including socioeconomic disadvantage [18] and SHCs [37], as well as resilience [23,29], self-efficacy [39,46], and physical activity levels [36]. For instance, a study from South Korea found that higher physical activity was associated with lower anxiety symptoms [42], highlighting physical activity as a potentially relevant factor to consider in future research.

Taken together, these results suggest a possible association between anxiety in SCI and physical complications, social context, and coexisting mental health conditions. However, compared with depression, anxiety remains comparatively underexplored in the SCI literature; in this review, anxiety outcomes were reported in only 15 included studies and were assessed using heterogeneous instruments and cut-off points, which limits comparability across studies. Further longitudinal research is warranted to clarify anxiety trajectories and long-term impact in this population.

PTSD and Suicidal Ideation

PTSD and suicidal ideation, while less frequently studied than depression or anxiety, were identified as significant mental health concerns following SCI. In the included studies, PTSD prevalence estimates primarily reflected screen-positive PTSD symptoms over a defined period and ranged from 12% to 36% [24,25]. Higher rates were reported among individuals with incomplete injury [24], socioeconomic hardship [18], decreased physical function, and limited coping resources [32,41]. Because WHO estimates describe lifetime PTSD prevalence in the general population (approximately 4%), these figures reflect a different time frame of assessment and are not directly comparable; nonetheless, the SCI literature indicates a notable burden of PTSD symptoms relative to general population levels [53].

Our findings on PTSD align with those of a previous systematic review by Pollock et al. [54], which reported that post-injury factors showed the strongest pooled associations with PTSD symptom severity. Specifically, depressed mood, negative appraisals, psychological distress, anxiety, and pain severity were consistently related to higher PTSD symptom severity. This corresponds with the present review, in which PTSD frequently co-occurred with depression and anxiety [25,32,41], and lower life satisfaction was also associated with greater PTSD symptom burden [24]. Taken together, both reviews suggest that psychological and somatic factors are commonly linked with PTSD symptom severity following SCI.

Suicidal ideation was reported in 11% to 33% of participants across the included studies, typically reflecting period prevalence [32,41]. It frequently co-occurred with depressive symptoms and was associated with low resilience and socioeconomic hardship [18,32,41]. By comparison, general population estimates are approximately 2%-3% annually and up to 9% over the lifetime [55]. Although these figures are not directly comparable because of differences in measurement approaches and reference periods, the available SCI literature nonetheless indicates a considerable burden of suicidal ideation. Interestingly, a systematic literature review from 2017 [56] noted that suicidal behavior following SCI was a frequent cause of death, with studies indicating that between 5.8% and 11% of deaths were attributable to suicide.

Resilience and social support were repeatedly associated with lower PTSD symptom severity and lower levels of suicidality, even among individuals experiencing pain or depressive symptoms [32,41]. Conversely, secondary health complications, social isolation, and financial stressors were associated with greater psychological distress [18,24,32]. The findings mentioned above underline the potential importance of integrating PTSD and suicide risk screening into SCI rehabilitation and follow-up care, alongside interventions that strengthen coping strategies and community connectedness to mitigate suicide risk.

Other Mental Health Outcomes

Evidence on alcohol and substance use disorders was limited. In one study of U.S. veterans included in this review, alcohol use disorder was reported in 8% of participants and substance use disorder in 5.3% [24]. Although only a single study addressed these outcomes, previous evidence suggests that such conditions represent an important yet underexplored aspect of mental health following SCI. This gap is further illustrated by findings from a comparative study by Graupensperger et al. [57], which revealed that, compared with non-SCI patients, individuals with SCI had significantly increased odds of alcohol use disorder, cannabis use disorder, opioid use disorder, and nicotine use disorder. Future research should clarify the mechanisms linking SCI and substance use-whether through pain management, coping strategies, or socioeconomic disadvantage-and develop tailored interventions to address this risk.

Factors Associated With Mental Health Outcomes After SCI

While individuals with SCI report consistently higher levels of depression, anxiety, PTSD symptoms, and suicidality than the general population, several psychosocial resources were consistently related to more favorable mental health outcomes across the included studies. Resilience was most frequently reported and was associated with fewer depressive symptoms, lower levels of anxiety, lower PTSD symptom severity, higher QoL, and lower suicidality, even in the presence of pain or SHCs [23,29,32,41,47]. Similarly, higher self-efficacy [39,46] was correlated with more favorable psychological adjustment, with greater confidence in managing one’s condition associated with lower psychological distress.

With respect to factors associated with risk, preinjury psychiatric history was examined in only one of the included studies; in that prospective cohort, a prior psychiatric disorder predicted persistent depressive symptoms after SCI [27], but this factor was rarely reported in the remaining studies. Older systematic reviews similarly note that premorbid factors such as psychiatric disorders and substance abuse increase the risk of psychological morbidity following SCI [52,56]. Future research should more consistently investigate pre-existing mental health history in individuals with SCI to clarify its role as a confounder in the association between SCI and mental health disorders.

It is noteworthy that the potentially predictive factors identified in this review align closely with those reported in previous literature. For example, Kraft and Dorstyn emphasized the role of psychosocial correlates, including resilience, social support, and self-efficacy, as key determinants of depression following SCI [58]. Similarly, Budd et al. highlighted resilience, adaptive coping, and social connectedness as central protective factors for psychological well-being [59], while van Leeuwen et al. demonstrated that changes in self-efficacy, mastery, and acceptance over time were strongly associated with mental health outcomes [60]. Together, these studies support the central role of psychosocial resources in shaping mental health trajectories after SCI.

Crucially, many of the mental health correlates appear to represent opposite ends of the same spectrum; for instance, higher resilience, stronger social support, and greater self-efficacy were associated with fewer depressive symptoms, whereas lower resilience, social isolation, and limited coping resources were associated with greater psychological vulnerability.

Social support from family, peers, or rehabilitation teams was also consistently associated with more favorable mental health outcomes, even in the context of functional limitations and socioeconomic hardship [31,38,43]. Studies further showed that participation in meaningful activities and higher levels of physical activity were linked to lower anxiety and depression scores [21,30,36,42], highlighting the relevance of community engagement and health-related behaviors in relation to psychological well-being.

Collectively, these findings suggest that these factors represent more than simple correlates and may constitute potentially modifiable targets for future intervention. Programs that integrate resilience-building, coping skills training, social network development, and participation-enhancing activities may therefore be relevant components of comprehensive rehabilitation approaches aimed at addressing psychological morbidity after SCI. Notably, these psychosocial resources appear to operate across diagnostic categories, being associated with multiple mental health outcomes rather than any single disorder in isolation.

Nevertheless, several methodological issues should be considered when interpreting these findings, particularly with respect to measurement validity, time frame, and cross-cultural adaptation. Most included studies used instruments that had been previously validated in individuals with SCI, and several reported using validated translations in the local language (e.g., Spanish, Polish, Turkish, and Nepali versions of standard depression and anxiety scales) [35,40,41,43]. However, formal evaluation of cross-cultural measurement invariance was rarely reported, so cross-country comparisons should be interpreted with caution. Notably, most studies reported period prevalence based on recent symptoms; fewer reported point or lifetime prevalence. This heterogeneity in time frame limits direct comparability across studies. In addition, many of these instruments include somatic items (e.g., sleep disturbance, fatigue, and appetite changes) that may overlap with common sequelae of SCI rather than reflecting psychopathology per se, raising the possibility of differential item functioning and misclassification. Moreover, future studies may benefit from focusing on more homogeneous populations with respect to cultural context, socioeconomic status, and access to mental health services, in order to facilitate interpretation and comparison across settings.

Clinical Implications

The findings of this review highlight the importance of placing mental health care at the core of SCI rehabilitation and long-term follow-up. The higher reported prevalence of depression, anxiety, PTSD, and suicidality compared with the general population underscores the relevance of routine screening for psychological distress in both inpatient and community settings. Validated, brief instruments such as the PHQ-9 [10], GAD-7 [11], and PTSD checklists (PCL-5) [16] may serve as efficient tools for early identification of clinically relevant symptoms.

In light of the factors associated with mental health outcomes mentioned above, rehabilitation programs may benefit from adopting a biopsychosocial approach that integrates physical and psychological care. Attention to pain, access to health services, and financial or vocational stability appear relevant in relation to mental health outcomes. Moreover, incorporating therapies such as cognitive behavioral therapy (CBT) into the care of individuals with SCI has been associated with improvements in anxiety and depressive symptoms in previous studies [61].

At the same time, the consistent associations observed for resilience, self-efficacy, and social support indicate that interventions targeting these domains may be useful adjuncts to standard rehabilitation. Scalable options include structured peer support groups, resilience training, and programs that encourage community participation and physical activity. These approaches are likely relevant across settings; however, implementation may be particularly challenging in low- and middle-income countries, where psychological distress appears more common and access to services is limited.

The importance of integrating psychological care into SCI rehabilitation has been emphasized for over a decade [52]. This literature supports moving beyond physical rehabilitation alone and embedding mental health screening and support within routine follow-up.

Strengths and Limitations

This review has several notable strengths. It synthesized evidence from 30 studies conducted across multiple countries and integrated a wide range of mental health outcomes, providing a broad overview of the psychological burden associated with SCI. Most included studies were of high quality, strengthening the reliability of the findings. In addition, the methodological quality of this narrative review was formally appraised using the SANRA [17], with an overall score of 10/12, indicating high methodological quality despite the inherent limitations of a non-systematic narrative approach.

Several limitations should also be acknowledged. First, the predominance of cross-sectional designs restricts causal inference, and the narrative (rather than systematic) review design raises the possibility of selection bias. In addition, our use of single-reviewer screening and data extraction with verification by a second reviewer, rather than full dual independent screening and extraction, may have increased the risk of selection and abstraction bias. Another limitation is that study quality was assessed using a seven-point checklist rather than design-specific, validated critical appraisal tools, which may have limited the precision of our risk-of-bias assessment across different study designs. Furthermore, the relatively narrow search strategy, which was restricted to core terms related to SCI and mental health, may have led to underidentification of eligible studies. We also did not conduct systematic citation tracking (snowballing) for all included studies; although we screened the reference lists of some key articles, this informal approach may have contributed to missing additional relevant studies. Considerable heterogeneity in study designs and outcome measures also precluded meta-analysis and limits confidence in any overall summary estimate. Finally, certain outcomes, including PTSD, suicidal ideation, and alcohol or substance use disorders, were investigated less frequently, limiting the conclusions that can be drawn in these areas.

## Conclusions

This narrative review highlights the profound mental health burden reported among individuals with SCI, with consistently higher rates of depression, anxiety, PTSD, and suicidal ideation compared with the general population. Factors such as pain interference, SHCs, and socioeconomic disadvantage were associated with greater vulnerability, whereas resilience, self-efficacy, social support, and active participation were associated with more favorable psychological outcomes. These findings underscore the importance of prevention, early identification, and targeted interventions for mental health concerns as integral components of SCI rehabilitation. By raising awareness of these challenges and highlighting potentially modifiable psychosocial resources, this review aims to inform clinical practice and research and to encourage the development of integrated care models and future studies addressing both the psychological and physical consequences of SCI.

## Appendices

Appendix A 

**Table 2:** Database search strategy (May 2025)

Database (platform)	Date searched	Search fields	Search terms	Limits/filters applied
PubMed	May 2025	PubMed default (all fields)	"spinal cord injury" AND "mental health"	Publication years 2015–2025; English; free full text/open access (as applied in PubMed filters)
Scopus	May 2025	Scopus basic search (as conducted)	"spinal cord injury" AND "mental health"	Publication years 2015–2025; English; open access; subject area limited to Medicine

Appendix B 

**Table 3:** Template of the structured data extraction form used in this review

Author (year)	Journal	Country	Study design	Sample size (n)	Mean age (years)	% male	Injury type (paraplegia/tetraplegia)	Time since injury	Mental health outcomes	Measurement tools	Outcome time frame/cut-off point(s)	Key findings	Study objective	Comparisons (if applicable)	Special cases (e.g., COVID-19 impact, veterans vs. general population)	Title

Appendix C 

**Table 4:** Characteristics of included studies and mental health outcome measurement definitions (author/year, outcome, methods/instruments, screening vs. diagnostic classification, time frame, cut-off point/case definition, study design) NR: not reported; GAD-7: Generalized Anxiety Disorder-7; PHQ-8: Patient Health Questionnaire-8; QoL: quality of life; MHI-5: Mental Health Inventory-5; HADS-D: Hospital Anxiety and Depression Scale-Depression subscale; PTSD: post-traumatic stress disorder; PC-PTSD: Primary Care PTSD Screen; WHOQoL-BREF: World Health Organization Quality of Life-BREF; SWLS: Satisfaction With Life Scale; PHQ-4: Patient Health Questionnaire-4; GAD-2: Generalized Anxiety Disorder-2; CES-D 10: 10-item Center for Epidemiologic Studies Depression Scale; STAI-T5: State-Trait Anxiety Inventory-Trait (5-item version); BDI: Beck Depression Inventory; STAI: State-Trait Anxiety Inventory; PCL-5: PTSD Checklist for Diagnostic and Statistical Manual of Mental Disorders, Fifth Edition; SI: suicidal ideation; GDS-15: 15-item Geriatric Depression Scale; DMMQ: Depressive Mood Management Questionnaire; SCI: spinal cord injury; SCI REHAB Project: Spinal Cord Injury Rehabilitation Project; PHQ-9: Patient Health Questionnaire-9; HADS: Hospital Anxiety and Depression Scale;

Author (year)	Mental health outcome	Methods and instruments for assessing outcomes	Screening or diagnostic	Outcome time frame	Cut-off point/case definition	Study design
1. Mikolajczyk et al. (2021) [23]	Depression/anxiety/resilience-QoL (during COVID-19 pandemic)	PHQ-8/GAD-7/SCI-QoL resilience scale	Screening (PHQ-8, GAD-7)	PHQ-8, GAD-7: past 2 weeks	PHQ-8 NR/GAD-7 NR	Cross-sectional ﬁndings from a larger longitudinal study
2. Zürcher et al. (2019) [38]	General mental health/depression	MHI-5/HADS-D	Screening	MHI-5: past 4 weeks, HADS-D: past week	MHI-5 ≥ 56; no general mental health problems/HADS-D ≥ 8; depressive symptomatology	Data analysis from the first cross-sectional community survey of the Swiss Spinal Cord Injury Cohort Study
3. McDonald et al. (2018) [24]	Depressive disorders/PTSD/alcohol use disorder/substance use disorders	Review of electronic medical records for diagnoses determined by team psychologist interviews. Diagnosis per the Diagnostic and Statistical Manual of Mental Disorders, Fourth Edition, Text Revision	Diagnostic	Point prevalence	NR	Cross-sectional
4. Kelly et al. (2023) [25]	Depression/anxiety/probable PTSD	PHQ-9 (excluding sleep item)/GAD-7/PC-PTSD	Screening	PHQ-9, GAD-7: past 2 weeks/PC-PTSD: past month	PHQ-9 ≥ 10; clinically relevant depressive symptoms/GAD-7 ≥ 10; clinically relevant anxiety symptoms/PC-PTSD-5 ≥ 3; indicative of probable PTSD	Cross-sectional
5. Bombardier et al. (2023) [26]	Depression/QoL	PHQ-9/WHOQoL-BREF	Screening	Past 2 weeks	PHQ-9 ≥ 10; moderate depression	Single blind parallel group randomized controlled trial (RCT)
6. Kim et al. (2020) [42]	Depression/anxiety	PHQ-9/GAD-7	Screening	Past 2 weeks	PHQ-9 ≥ 10; clinically relevant depressive symptoms/GAD-7 ≥ 10; moderate anxiety, clinically relevant symptoms	Cross-sectional
7. Bombardier et al. (2016) [27]	Depression	PHQ-9	Screening	Past 2 weeks (assessed at 4 different time points)	PHQ-9 ≥ 10 clinically relevant depressive symptoms	Longitudinal cohort study
8. Richardson et al. (2016) [28]	Satisfaction in life among depressed individuals with SCI (PHQ-9 ≥ 10)	SWLS	SWLS is a measure of an individual’s subjective global judgment of their life	After 12 weeks	NR	Cross-sectional/longitudinal components using data from an existing RCT
9. Vives Alvarado et al. (2022) [29]	Depression/anxiety/resilience-satisfaction with life (during COVID-19 pandemic)	PHQ-9/GAD-7/SWLS	PHQ-9, GAD-7: screening	PHQ-9, GAD-7: past 2 weeks	PHQ-9 NR/GAD-7 NR	Cohort telephone survey study
10. Graupensperger et al. (2021) [22]	Anxiety/depressive disorders	International Classification of Diseases (ICD)	Diagnostic	NR	NR	Retrospective cohort study/cross-sectional
11. Mulroy et al. (2016) [30]	Depression/satisfaction with life	PHQ-2/SWLS	PHQ-2: screening	PHQ-2: past 2 weeks	PHQ-2 ≥ 3, clinically relevant depressive symptoms	Cross-sectional
12. Watson et al. (2022) [31]	Depression/anxiety	PHQ-4 (PHQ-2, GAD-2)	Screening	NR	PHQ-4 ≥ 6, higher total score indicates greater mental health symptom severity	Cross-sectional
13. Williams et al. (2024) [45]	Depression/anxiety/life satisfaction	CES-D 10/STAI-T5/WHOQoL-BREF	Screening	NR	NR	Cross-sectional
14. Fekete et al. (2022) [19]	Mental health	MHI-5	Screening	NR	MHI-5 NR	Cross-sectional
15. Molina-Gallego et al. (2024) [35]	Anxiety/depression	BDI/STAI	Screening	BDI: last 2 weeks	BDI > 17; indicative of moderate depression/STAI > 39–40; detecting clinically significant symptoms	Cross-sectional
16. Usta Sağlam et al. (2023) [41]	PTSD/depression/SI/resilience	PCL-5/PHQ-9/Brief Resilience Scale	Screening	NR	Turkish version PCL-5 ≥ 47; suggested for screening of PTSD/PHQ-9; scores of 5, 10, 15, and 20 serve as cut-off scores indicating mild, moderate, moderately severe, and severe depression/9th item of PHQ-9 ≥ 1 indicative of having SI	Cross-sectional
17. Jörgensen et al. (2017) [36]	Depressive symptoms	GDS-15	Screening	NR	GDS-15 ≥ 5; clinically relevant depressive symptoms; ≥10 indicates probable depression	Cross-sectional
18. Sanguinetti et al. (2022) [18]	Mood and anxiety disorders/depression/suicidal thoughts/satisfaction with life	Mental health assessed using standardized survey modules. Data from Canadian Community Health Survey 2010	NR	NR	NR	Retrospective cross-sectional epidemiological study using data from the 2010 Canadian Community Health Survey
19. Craig et al. (2015) [47]	Depression/self-efficacy	HADS/psychological disorders assessed by the Mini International Neuropsychiatric Interview	Screening	Past 2 weeks	HADS: NR	Prospective cohort
20. Joseph et al. (2023) [37]	Depression/anxiety/general psychological distress	SF-36 version 2	NR	NR	NR	Cross-sectional
21. van Diemen et al. (2021) [39]	Depression/anxiety/self-efficacy	HADS/University of Washington Self-Efﬁcacy Scale	Screening	NR	HADS NR	Multi-center longitudinal inception cohort study
22. Craig et al. (2019) [46]	Depressive mood/self-efficacy	HADS/Moorong Self-Efficacy Scale for self-efficacy	NR	NR	HADS-D NR	Prospective cohort
23. Baniya et al. (2019) [43]	Depressive mood	PHQ-9/DMMQ	NR	NR	PHQ-9 NR	Cross-sectional
24. Khong et al. (2023) [32]	Suicidal ideation/depression/anxiety/resilience-satisfaction with life	PHQ-9/SWLS/SCI-QoL short form	Screening	PHQ-9: past 2 weeks	PHQ-9; using item 9 for SI, ≥1 indicative of SI	Cross-sectional
25. Müller et al. (2017) [21]	Depressive symptoms/QoL	MHI-5/WHOQoL-BREF	NR	NR	MHI-5 NR	Cross-sectional
26. Makkar et al. (2025) [44]	Psychological well-being and QoL	GHQ-12/WHOQoL-BREF	NR	NR	GHQ-12 > 15 indicate distress; >20 indicate severe psychological distress	Cross-sectional
27. Clark et al. (2025) [33]	Depression	PHQ-9	Screening	NR	PHQ-9 NR	Longitudinal analyses of self-report assessment data
28. Parker et al. (2022) [20]	Depression/anxiety/life satisfaction	PHQ-9/GAD-2/SWLS	NR	NR	PHQ-9 ≥ 10; moderately severe depressive symptoms/GAD-2 ≥ 2; indicated clinically signiﬁcant anxiety	Cohort (SCIRehab Project)
29. Widuch-Spodyniuk et al. (2023) [40]	Depression/anxiety	Depression Assessment Questionnaire (KPD)/STAI-Polish version	NR	NR	KPD > 130; suggesting depressive disorder/STAI NR	Prospective clinical study
30. Boals et al. (2017) [34]	Post-traumatic stress symptoms/depressed mood	PC-PTSD/PHQ-8	PHQ-8, PC-PTSD: screening	PHQ-8: past two weeks	NR	Cross-sectional

Appendix D 

**Table 5:** SANRA: quality assessment of this narrative review Rating scale of each item: 0 = not fulfilled; 1 = partially fulfilled; 2 = fully fulfilled Total score range: 0-12 Total score: 10-12 → high quality Total score: 7-9 → moderate quality Total score: 0-6 → low quality SANRA: Scale for the Assessment of Narrative Review Articles [17]; SCI: spinal cord injury; WHO: World Health Organization

SANRA items	Rating (0–2)	Comments
1. Justification of the article’s importance for the readership	2	Importance of the burden in mental health after SCI clearly explained.
2. Statement of concrete aims or formulation of questions	2	Aim of synthesizing evidence on the impact of SCI on mental health.
3. Description of the literature search	1	Search strategy described; some limitations acknowledged.
4. Referencing (completeness and appropriateness)	2	Key statements and studies and WHO data appropriately cited.
5. Scientific reasoning	1	Narrative synthesis with critical interpretation and integration, but non-systematic.
6. Appropriate presentation of data	2	Results structured by outcome domain and tables used.
Total score	10/12	Indicating a methodologically high-quality narrative review.

Appendix E 

**Table 6:** Quality assessment of the included studies using the seven-domain checklist

30 studies (author, year)	Clear objective	Appropriate study design	Population description	Measurement quality	Data analysis clarity	Bias & confounding consideration	Transparency of funding & conflicts	Total score out of 7
Mikolajczyk et al. (2021) [23]	Yes	Yes	Yes	Yes	Partial	Yes	Yes	6, high quality
Zürcher et al. (2019) [38]	Yes	Yes	Yes	Yes	Yes	Yes	Yes	6, high quality
McDonald et al. (2018) [24]	Yes	Yes	Yes	Partial	Yes	Partial	Yes	5, moderate quality
Kelly et al. (2023) [25]	Yes	Yes	Yes	Yes	Yes	Partial	Yes	6, high quality
Bombardier et al. (2023) [26]	Yes	Yes	Yes	Yes	Yes	Yes	Yes	7, high quality
Kim et al. (2020) [42]	Yes	Yes	Yes	Yes	Yes	Partial	Yes	6, high quality
Bombardier et al. (2016) [27]	Yes	Yes	Yes	Yes	Yes	Partial	Yes	6, high quality
Richardson et al. (2016) [28]	Yes	Yes	Yes	Yes	Yes	Partial	Yes	6, high quality
Vives Alvarado et al. (2022) [29]	Yes	Yes	Partial	Yes	Yes	Partial	Yes	5, moderate quality
Graupensperger et al (2021) [22]	Yes	Yes	Partial	Yes	Yes	Partial	Yes	5, moderate quality
Mulroy et al. (2016) [30]	Yes	Yes	Yes	Yes	Yes	Partial	Yes	6, high quality
Watson et al. (2022) [31]	Yes	Yes	Yes	Yes	Yes	Partial	Yes	6, high quality
Williams et al. (2024) [45]	Yes	Yes	Yes	Yes	Yes	Partial	Partial	5, moderate quality
Fekete et al. (2022) [19]	Yes	Yes	Yes	Yes	Yes	Yes	Yes	7, high quality
Molina-Gallego et al. (2024) [35]	Yes	Yes	Yes	Yes	Yes	Partial	Partial	5, moderate quality
Usta Sağlam et al. (2023) [41]	Yes	Yes	Yes	Yes	Yes	Partial	Yes	6, high quality
Jörgensen et al. (2017) [36]	Yes	Yes	Yes	Yes	Yes	Partial	Yes	6, high quality
Sanguinetti et al. (2022) [18]	Yes	Yes	Yes	Yes	Yes	Yes	Yes	7, high quality
Craig et al. (2015) [47]	Yes	Yes	Yes	Yes	Yes	Yes	Yes	7, high quality
Joseph et al. (2023) [37]	Yes	Yes	Yes	Yes	Yes	Partial	Yes	6, high quality
van Diemen et al. (2021) [39]	Yes	Yes	Yes	Yes	Yes	Yes	Yes	7, high quality
Craig et al. (2019) [46]	Yes	Yes	Yes	Yes	Yes	Partial	Yes	6, high quality
Baniya et al. (2019) [43]	Yes	Yes	Yes	Partial	Yes	Partial	Yes	5, moderate quality
Khong et al. (2023) [32]	Yes	Yes	Yes	Yes	Yes	Partial	Yes	6, high quality
Müller et al. (2017) [21]	Yes	Yes	Yes	Yes	Yes	Partial	Yes	6, high quality
Makkar et al. (2025) [44]	Yes	Yes	Yes	Yes	Yes	Partial	Yes	6, high quality
Clark et a. (2025) [33]	Yes	Yes	Yes	Yes	Yes	Yes	Yes	7, high quality
Parker et al. (2022) [20]	Yes	Yes	Yes	Yes	Yes	Yes	Yes	7, high quality
Widuch-Spodyniuk et al. (2023) [40]	Yes	Partial	Yes	Yes	Yes	Partial	Yes	5, moderate quality
Boals et al. (2017) [34]	Yes	Yes	Yes	Yes	Yes	Partial	Yes	6, high quality
